# HHV-6A Drives Epigenetic Reprogramming via an EZH2–SIRT1 Axis to Sustain Mutant p53 and Reshape Oncogenic Inflammatory Signaling

**DOI:** 10.3390/v18040409

**Published:** 2026-03-26

**Authors:** Rossella Benedetti, Michele Di Crosta, Alessia Stirparo, George Alexandru Aron, Stefania Mardente, Roberta Santarelli, Roberta Gonnella, Maria Saveria Gilardini Montani, Mara Cirone

**Affiliations:** Department of Experimental Medicine, Sapienza University, 00161 Rome, Italy

**Keywords:** HHV-6A, EZH2, SIRT1, mutp53, c-Myc, IL-6

## Abstract

We previously demonstrated that human herpesvirus 6A infects papillary thyroid cancer cells (BCPAP), inducing molecular changes compatible with a tumor-promoting phenotype, including increased expression of R273H mutant TP53 (mutp53), upregulation of c-Myc, and enhanced secretion of IL-6. To investigate whether and how epigenetic mechanisms contribute to these virus-induced effects, we examined the histone methyltransferase EZH2, a key regulator of chromatin repression frequently altered in cancer. HHV-6A infection reduced EZH2 expression and global H3K27me3 levels. Pharmacological inhibition of EZH2 using DS-3201 reproduced some of the molecular effects of viral infection, including increased mutp53 stability. Both viral infection and EZH2 inhibition induced delayed upregulation of SIRT1, which mediated deacetylation-dependent stabilization of mutp53 while reducing c-Myc expression. Indeed, the inhibition of SIRT1 with EX-527 reversed mutp53 accumulation but restored c-Myc expression and increased extracellular IL-6 release. This drug also reduced cell survival, suggesting that SIRT1 supports cellular adaptation to oncogenic stress triggered by EZH2 loss. Overall, our findings identify an epigenetic axis in which the HHV-6A-mediated downregulation of EZH2 induces SIRT1, regulating mutp53 stability and c-Myc expression and reshaping inflammatory signaling to maintain cell viability. These results establish a mechanistic link between viral infection, epigenetic remodeling, and oncogenic dependency. They also suggest that targeting IL-6 signaling could represent a therapeutic vulnerability in HHV-6A-associated thyroid cancer, particularly in combination with SIRT1 inhibitors.

## 1. Introduction

Viral infection frequently induces widespread changes in DNA methylation patterns, histone modifications, and non-coding RNA expression, thereby altering cellular and viral gene expression without modifying the underlying DNA sequence. These epigenetic alterations not only facilitate viral persistence and immune evasion but may also create a cellular environment conducive to disease onset [1]. Viral-driven epigenetic and genomic perturbations may initiate malignant transformation on their own or contribute to establishing a permissive environment that cooperates with other stimuli to promote tumor progression [2]. A better understanding of the interplay between viruses and the host epigenetic machinery could provide critical insights into the molecular mechanisms of virus-associated diseases and open new avenues for targeted therapeutic interventions and biomarker discovery.

Human herpesvirus 6 (HHV-6) A is a ubiquitous β-herpesvirus that, similarly to other herpesviruses, can establish lifelong latency in the host. It has been implicated in a broad range of neurological, autoimmune, and inflammatory disorders [3,4,5]. Although HHV-6 cannot be classified as a canonical oncogenic virus, accumulating evidence suggests that it may indirectly contribute to tumorigenesis [6]. Indeed, the HHV-6A ’s ability to integrate into human chromosomal telomeres and reactivate under certain conditions raises intriguing questions about its long-term effects on genomic stability [6,7].

Moreover, HHV-6A and B may modulate the host cell epigenome by inducing DNA hypomethylation in the regions close to telomeres and can enrich the viral genome with repressive histone marks H3K9me3/H3K27me3 during latency [7,8].

In previous studies, it has been reported that HHV-6A can be detected in thyroid tissues, particularly in association with autoimmune inflammatory conditions [5]. Interestingly, epigenetic remodeling may contribute to the activation of pro-inflammatory pathways, including Nuclear Factor kappa-light-chain-enhancer of activated B cells (NF-κB), Signal Transducer and Activator of Transcription 3 (STAT3), and Activating Protein-1 (AP-1), and the signaling mediated by these transcription factors may lead to tissue damage and genomic instability, thus favoring cellular transformation. We recently reported that human papillary tumor cells (BCPAP) could be infected by HHV-6A and that viral infection induced several changes compatible with a tumor-promoting phenotype [9], which, in the case of this low-grade thyroid tumor, may be the progression into the follicular carcinoma (FTC) and the anaplastic carcinoma (ATC). Among the pro-tumorigenic effects, HHV-6A upregulated R273H mutp53 (mutp53) and c-Myc and increased the release of interleukin-6 (IL-6) [9], a cytokine that, besides creating a pro-inflammatory/oncogenic environment, can act as a growth factor for thyroid tumor cells [10]. Based on this background, here we investigated whether HHV-6A infection could induce epigenetic modifications that underlie these pro-tumorigenic effects previously observed in BCPAP cells. We focused on Enhancer of zeste homolog 2 (EZH2), a histone methyltransferase that regulates chromatin structure and gene expression through the addition of repressive mark H3K27me3, as it is frequently dysregulated in cancer and may contribute to the epigenetic silencing of tumor suppressor genes, promoting cell proliferation [11]. Altered expression or activity of this enzyme is also linked to chronic inflammation [12], inflammation-driven carcinogenesis, and virus-induced epigenetic reprogramming [13], highlighting its central role at the intersection of infection, inflammation, and cancer. Recent evidence indicates that EZH2 may not act in isolation, but its activity can be interconnected with Sirtuin 1 (SIRT1) [14]. As both of them are components of an epigenetic regulatory network governing chromatin repression and cellular stress responses, and may induce post-translational modification (PTMs) on non-histone proteins, altering the stability and function of transcription factors such as c-Myc and p53 [15,16], we investigated whether this network could be exploited by HHV-6A infection to sustain BCPAP tumorigenicity.

## 2. Materials and Methods

### 2.1. Cell Cultures, Infection, and Treatments

Papillary thyroid carcinoma BCPAP cell line, obtained from ECACC, and thyroid anaplastic carcinoma CAL-62 cell line, obtained from ATCC, were maintained at 37 °C in a humidified atmosphere with 5% CO_2_ and cultured in RPMI 1640 medium (PAN-Biotech, Adenbach, Germany) supplemented with 10% fetal bovine serum (FBS) (Sial, Rome, Italy), L-glutamine (100 μg/mL; Aurogene, Rome, Italy, AU-X0550-100), and penicillin–streptomycin (100 U/mL; Aurogene, Rome, Italy, AU-L0022-100). HHV-6A was propagated in HSB-2 cells as previously described [9]. BCPAP cells were seeded in 12-well plates at a density of 100,000 cells/well, while CAL-62 cells were seeded at a density of 50,000 cells/well. On the following day, cells were infected with an appropriate dilution of the HHV-6A viral stock (approximately 7 × 10^7^ viral DNA copies) in 0.2 mL of medium. After incubation for 1 h at 37 °C, 0.8 mL of complete medium was added, and cultures were maintained for the indicated times. Mock-infected controls were treated with heat-inactivated virus stock filtered through a 0.22 µm pore-size filter. Alternatively, BCPAP or CAL-62 cells were treated with valemetostat (DS-3201), an EZH2 inhibitor (Selleckchem, Houston, TX, USA, S8926), at a final concentration of 10 µM, or with MS177 an EZH2 PROTAC degrader (Sigma-Aldrich, St. Louis, MO, USA, SML3539), at a final concentration of 1 µM. In some experiments, the SIRT1 inhibitor selisistat (EX-527) (MedChemExpress, Monmouth Junction, NJ, USA, HY-15452) was used at 100 µM as a 1 h-pretreatment and was maintained throughout HHV-6A infection or DS-3201 treatment.

### 2.2. Quantitative Real-Time Polymerase Chain Reaction (qRT-PCR)

60 h after infection, BCPAP cells were washed with phosphate-buffered saline (PBS) and detached using 0.25% trypsin–EDTA. To remove non-internalized virus and extracellular viral DNA, cells were treated with DNase I (1 U/µL; Norgen Biotek Corp., Thorold, ON, Canada) for 30 min at 37 °C. Genomic DNA was then extracted using the ELITe Galaxy system (ELITechGroup S.p.A., Turin, Italy) according to the manufacturer’s instructions.

The presence of HHV-6 DNA was assessed by TaqMan real-time PCR using a commercially available kit targeting the ORF 13R (U67) gene region (ELITechGroup S.p.A., Turin, Italy) and analyzed on an ABI 7300 Real-Time PCR System (Applied Biosystems, Waltham, MA, USA).

Following infection or treatments, total RNA was isolated using TRIzol™ Reagent (Invitrogen, Waltham, MA, USA, 15596026). Reverse transcription was performed using the SensiFAST cDNA Synthesis Kit (Meridian, Cincinnati, OH, USA, BIO-65054), and qRT-PCR analysis was carried out with the SensiFAST SYBR Lo-ROX Kit (Meridian, Cincinnati, OH, USA, BIO-94020). Pre-designed KiCqStart^®^ SYBR^®^ Green RT-qPCR primers for *EZH2*, *TP53*, *SIRT1,* and *B2M* were purchased from Sigma-Aldrich (St. Louis, MO, USA, KSPQ12012). *c-MYC* mRNA expression level was analyzed using TaqMan gene expression assays (Applied Biosystems, Waltham, MA, USA, c-MYC: Hs00153408_m1, reference gene B2M: HS99999907-m1).

### 2.3. Cell Viability Assay

After treatments, a trypan blue dye (Sigma-Aldrich, St. Louis, MO, USA, T8154) exclusion assay was performed to determine the number of viable cells. Cells were counted by light microscopy using a Neubauer hemocytometer (Brand, Wertheim, Germany). The experiments were performed in triplicate and repeated at least three times.

### 2.4. Western Blot Analysis

Whole-cell protein lysates were prepared as previously described [17]. Briefly, cells were washed with PBS, lysed in RIPA buffer, and centrifuged at 14,000 rpm for 20 min. Protein concentrations were determined using the Bio-Rad Protein Assay (Bio-Rad, Hercules, CA, USA, 5000206), and equal amounts of protein were resolved on 4–12% NuPAGE Bis-Tris gels (Life Technologies, Carlsbad, CA, USA, N00322BOX).

Proteins were transferred onto nitrocellulose membranes (Amersham, Wilmington, DE, USA, 10600003) for 1 h in Tris–glycine buffer. Membranes were then blocked in PBS containing 0.1% Tween-20 and 3% BSA, incubated with the appropriate primary antibodies, and developed using ECL Blotting Substrate (Advansta, San Jose, CA, USA, K-12045-D20). Chemiluminescent signals were detected with a ChemiDoc Imaging System (Bio-Rad, Hercules, CA, USA).

The following primary antibodies were used: rabbit polyclonal anti-EZH2 (Proteintech, Rosemont, IL, USA, 21800-1-AP); mouse monoclonal anti-H3K27me3 (Sigma-Aldrich, 05-1951-S); mouse monoclonal anti-p53 (clone DO-1; Santa Cruz Biotechnology, Dallas, TX, USA, sc-126); rabbit polyclonal anti-IL-6 (Proteintech, 21865-1-AP); rabbit polyclonal anti-acetyl-p53 (Lys382) (St. John’s Laboratory, London, UK, ST598865-20); mouse monoclonal anti-p300 (Santa Cruz Biotechnology, sc-48343); mouse monoclonal anti-TIP60 (Santa Cruz Biotechnology, sc-166323); mouse monoclonal anti-SIRT1 (Proteintech, 60303-1-Ig); mouse monoclonal anti-p-STAT3 (Santa Cruz Biotecnology, B-7, sc-8059); rabbit polyclonal anti- STAT3 (Santa Cruz Biotecnology, C-20, sc-482); rabbit polyclonal anti-PTPN6 (Proteintech, 24546-1-AP); mouse monoclonal anti-c-Myc (Proteintech, 67447-1-Ig); and mouse monoclonal anti-PARP (Proteintech, 66520-1-Ig). Mouse monoclonal anti-β-Actin (Sigma-Aldrich, A5316) and mouse monoclonal anti-GAPDH (Santa Cruz Biotecnology, sc-32233) were used as a loading control. HRP-conjugated goat anti-mouse (Sigma-Aldrich, 401215) and goat anti-rabbit (Sigma-Aldrich, DC03L) antibodies were used as secondary antibodies. All antibodies were diluted in PBS containing 0.1% Tween-20 and 3% BSA.

### 2.5. Chemiluminescent Immunometric Assay (Luminex Assay)

After treatments, supernatants from BCPAP cells were collected, and interleukin-6 (IL-6) release was measured via a magnetic Luminex assay, using a human pre-mixed multi-analyte kit (R&D systems Bio-Techne, Minneapolis, MN, USA, LXSAHM), according to the manufacturer’s instructions.

### 2.6. Densitometric Analysis

Protein band intensities were quantified by densitometric analysis using Image Lab software v6.1 (Bio-Rad, Hercules, CA, USA).

### 2.7. Statistical Analysis

The results are represented as the mean ± standard deviation (S.D.) of at least three independent experiments, and statistical analyses were performed with Graphpad Prism software (v10.4.2). The two-tailed Student *t* test or a nonparametric 1-way analysis of variance (ANOVA) test was used to demonstrate statistical significance. Difference was considered statistically significant when *p* values were at least <0.05.

## 3. Results

### 3.1. HHV-6A Infection of BCPAP Downregulates EZH2 and Induces a Global Reduction of H3K27me3

**Figure 1:**
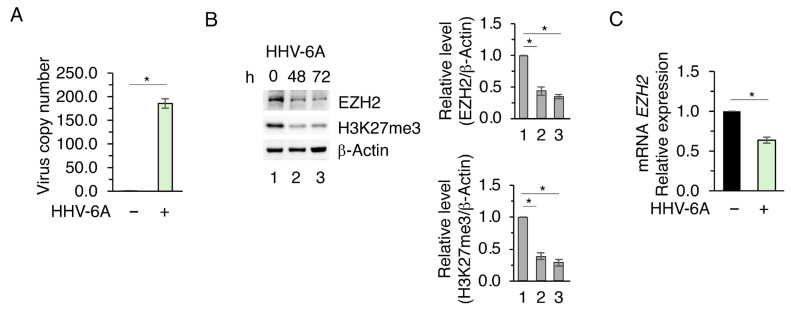
HHV-6A infection downregulates EZH2 expression in BCPAP cells. (**A**) Viral copy number determined by qRT-PCR in mock-treated and HHV-6A–infected cells 60 h post-infection. (**B**) Western blot analysis showing EZH2 and H3K27me3 protein levels in HHV-6A–infected BCPAP cells for the indicated times. β-Actin was used as a loading control. Densitometric analyses were normalized to β-Actin and expressed as fold change relative to control. (**C**) qRT-PCR analysis of *EZH2* mRNA expression in mock- and HHV-6A–infected BCPAP cells 72 h post-infection. Data were normalized to the reference gene B2M and expressed relative to the control. All data are shown as mean ± S.D. from three independent experiments. *p* value * < 0.05.

We have shown that HHV-6A infects the tumor papillary thyroid cancer cells (BCPAP), leading to several effects compatible with tumor progression into more aggressive forms of thyroid cancer. Indeed, viral infection resulted in an increased mutp53 expression level, a higher IL-6 secretion, and the upregulation of c-Myc in comparison to mock-infected cells [9]. Here, we investigated whether HHV6-A could induce epigenetic changes in BCPAP that could contribute to these effects. We focused on EZH2, as this methyltransferase is often dysregulated by viral infection and may play an important role in carcinogenesis [18]. After confirming HHV-6A infection of BCPAP by performing qPCR (Figure 1A), we observed that EZH2 expression level was reduced by HHV-6A infection and was accompanied by a global reduction in H3K27me3, a repression mark positively regulated by EZH2 (Figure 1B). Such downregulation was dependent on a reduced mRNA expression following viral infection (Figure 1C). These results suggest that HHV-6A infection dysregulates the expression of an epigenetic enzyme involved in the negative control of gene transcription, altering the epigenetic landscape in BCPAP.

### 3.2. HHV-6A and EZH2 Inhibitor Valemetostat (DS-3201) Stabilize Mutp53 Through SIRT1-Upregulation

**Figure 2:**
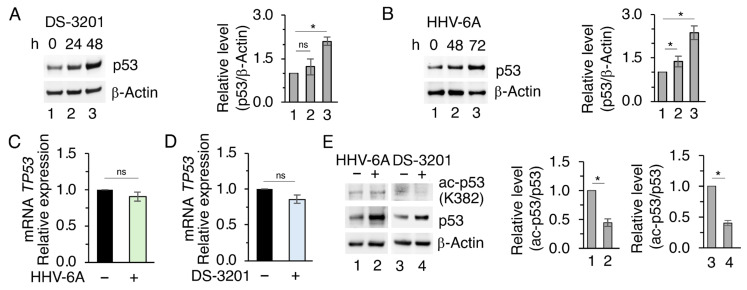
HHV-6 infection and EZH2 inhibition deacetylate and stabilize mutp53. (**A**,**B**) Western blot analysis of mutp53 protein level in BCPAP treated with the EZH2 inhibitor DS-3201 or infected by HHV-6A for the indicated times. (**C**,**D**) qRT-PCR analysis of *TP53* mRNA level in BCPAP cells mock- or HHV-6A-infected for 72 h or treated with DS-3201 for 48 h. Data were normalized to *B2M* and expressed relative to the control. (**E**) Western blot analysis of acetyl-p53 (K382) and mutp53 protein levels in BCPAP cells mock- or HHV-6A-infected for 72 h or treated with DS-3201 for 48 h. Densitometric analyses were normalized to the appropriate control and expressed as fold change relative to the untreated condition. All data are shown as mean ± S.D. from three independent experiments. *p* value * < 0.05, ns: non-significant.

**Figure 3:**
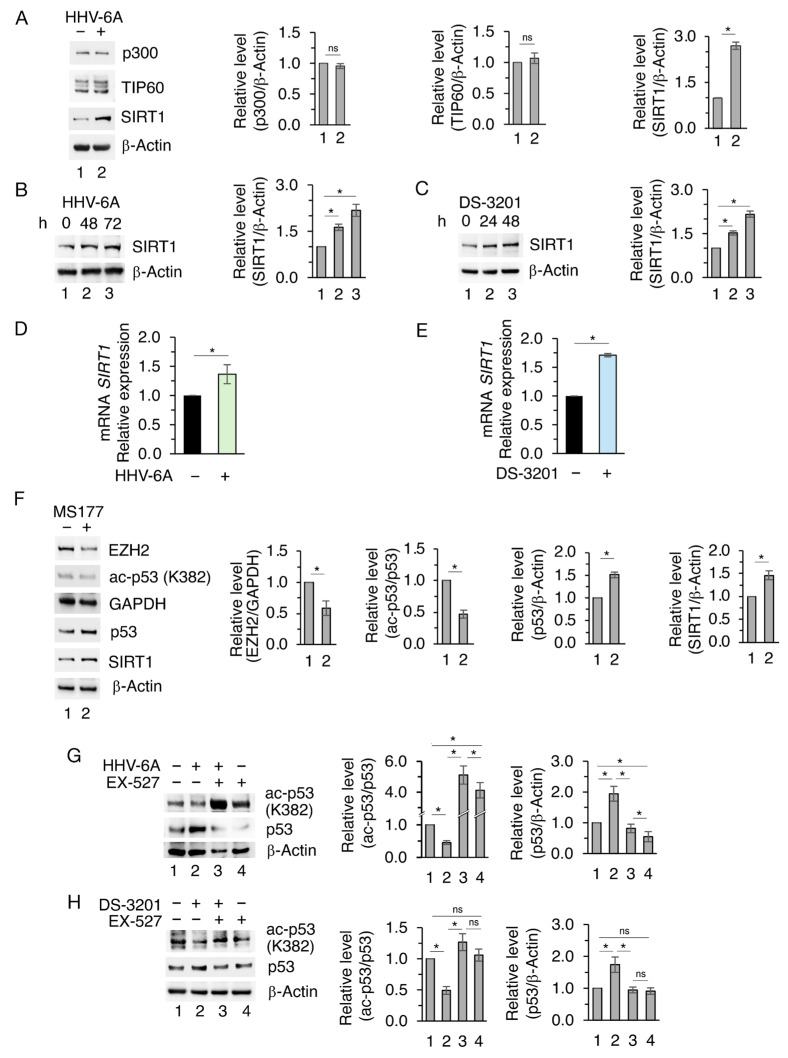
HHV-6 infection and EZH2 inhibition upregulate SIRT1, leading to mutp53 deacetylation and stabilization. (**A**) Western blot analysis of p300, TIP60, and SIRT1 protein levels in BCPAP cells mock- or HHV-6A-infected for 72 h. (**B**,**C**) Western blot analysis of SIRT1 protein levels at the indicated times in BCPAP cells HHV-6A-infected or treated with DS-3201. (**D**,**E**) qRT-PCR analysis of *SIRT1* mRNA levels in BCPAP cells mock- or HHV-6A-infected for 72 h or treated with DS-3201 for 48 h. Data were normalized to the reference gene B2M and expressed relative to the control. (**F**) Western blot analysis of EZH2, acetyl-p53 (K382), mutp53, and SIRT1 protein levels in BCPAP cells treated with the EZH2 PROTAC degrader MS177 for 48 h. (**G**,**H**) Western blot analysis of acetyl-p53 (K382) and mutp53 protein levels in BCPAP cells pre-treated or not with the SIRT1 inhibitor EX-527 for 1 h and subsequently mock- or HHV-6A-infected for 72 h or treated with DS-3201 for 48 h. β-Actin or GAPDH was used as a loading control. Densitometric analyses were normalized to the appropriate control and expressed as fold change relative to the untreated condition. All data are shown as mean ± S.D. from three independent experiments. *p* value * < 0.05, ns: non-significant.

To evaluate the impact of EZH2 downregulation on the upregulation of mutp53 induced by HHV-6A infection, we treated BCPAP with the EZH2-specific inhibitor valemetostat (DS-3201). As EZH2 expression was not affected during the first 24 h of HHV-6A infection, we treated BCPAP with DS-3201 for 24 h and 48 h and compared the effects with those induced in BCPAP after 48 h and 72 h of infection. We observed that DS-3201 upregulated mutp53 in a time-dependent fashion (Figure 2A) similar to HHV-6A infection (Figure 2B), suggesting that EZH2 inhibition was involved in the upregulation of mutp53. Next, to assess whether the increased expression of mutp53 could occur at the transcriptional level, qRT-PCR was performed to evaluate mRNA expression. As shown in Figure 2C,D, mutant *TP53* mRNA was slightly affected by these treatments, suggesting a post-transcriptional regulation of this protein following EZH2 inhibition. Searching for possible mechanisms leading to mutp53 upregulation in HHV-6A-infected or DS-3201-treated BCPAP, we investigated if changes in mutp53 acetylation could occur, as this PTM, particularly affecting lysine K382, may strongly influence mutp53 stability [19,20,21,22]. The results shown in Figure 2E indicate that mutp53 acetylation at K382 lysine residue was reduced by HHV-6A infection as well as by DS-3201 treatment, which may contribute to increased mutp53 stability. Indeed, previous studies have shown that increased acetylation of some p53 mutant proteins may reduce their protein stability [19,20]. We then evaluated if the expression of acetyltransferases (HATs) such as p300 and TIP60 or deacetylases such as SIRT1, known to regulate p53 acetylation [23,24], could be modified by HHV-6A infection in BCPAP and be involved in mutp53 deacetylation. We found that the expression of the above-reported HATs was slightly affected, while the expression level of SIRT1 increased in HHV-6A-infected BCPAP (Figure 3A). We then observed that the upregulation of SIRT1, either induced by HHV-6A infection or by DS-3201 treatment, was time dependent (Figure 3B,C). Moreover, both treatments also increased *SIRT1* mRNA, as evaluated by qRT-PCR (Figure 3D,E). Similar results in terms of acetyl and total mutp53 and SIRT1 were obtained by treating BCPAP with EZH2 PROTAC MS177, which was able to reduce the expression level of this methyltransferase (Figure 3F).

The role of SIRT1 on mutp53 changes was then demonstrated by using EX-527, a drug that was able to counteract mutp53 acetylation and accumulation (Figure 3G,H).

### 3.3. The Inhibition of SIRT1 Enhances the Release of IL-6 Without Activating STAT3 in HHV-6A-Infected or DS-3201-Treated Cells

**Figure 4:**
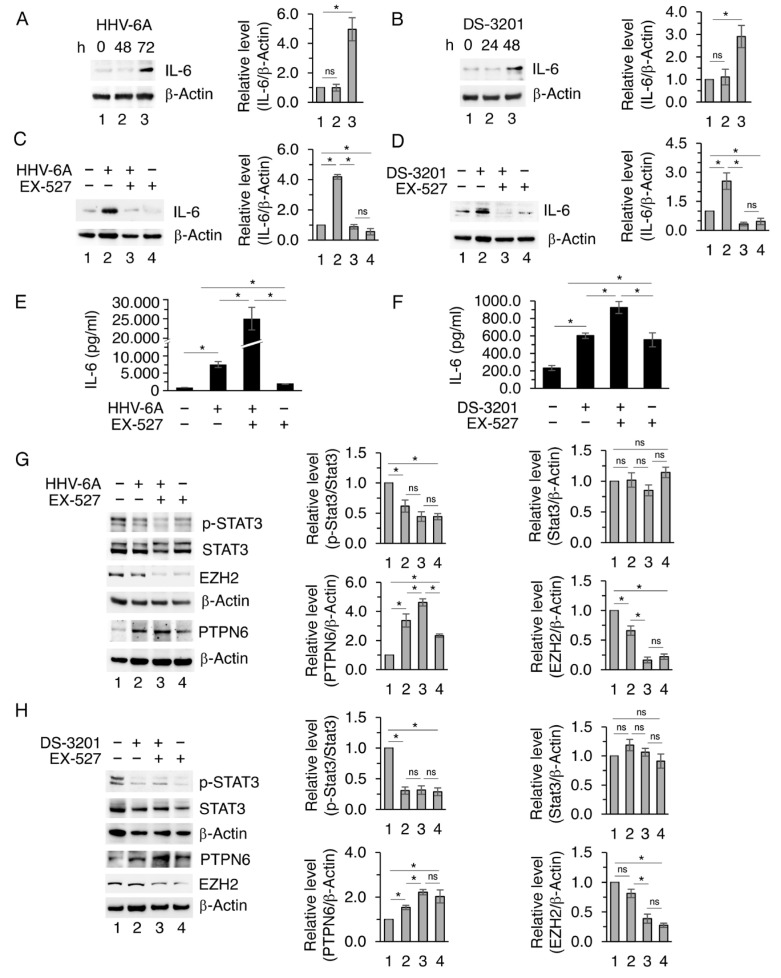
IL-6 release increases following SIRT1 inhibition, while PTPN6 maintains STAT3 in an unphosphorylated state. (**A**,**B**) Western blot analysis of IL-6 protein level in BCPAP cells infected by HHV-6A or treated with DS-3201 for the indicated times. (**C**–**H**) BCPAP cells were pre-treated or not with the SIRT1 inhibitor EX-527 for 1 h and subsequently mock- or HHV-6A-infected for 72 h or treated with DS-3201 for 48 h, then IL-6 (**C**,**D**), p-STAT3, STAT3, PTPN6, and EZH2 (**G**,**H**) protein levels were evaluated by Western blot analysis. β-Actin was used as a loading control. Densitometric analyses were normalized to the appropriate control and expressed as fold change relative to the untreated condition. IL-6 release in the supernatants was evaluated by Luminex assay (**E**,**F**). All data are shown as mean ± S.D. from three independent experiments. *p* value * < 0.05, ns: non-significant.

The impact of EZH2 and SIRT1 on IL-6, whose release was previously shown to increase following HHV-6A infection, was investigated by using DS-3201 in the absence or in the presence of EX-527 SIRT1 pharmacological inhibitor [25]. We found that HHV-6A infection, as well as DS-3201 treatment, upregulated IL-6 (Figure 4A,B), and that EX-527 reduced IL-6 expression level (Figure 4C,D). Similarly, EX-527 downregulated IL-6 expression level in EZH2 PROTAC MS177-treated BCPAP (Supplementary Figure S1A). The reduction in IL-6 could correlate with the reduction in mutp53 induced by EX-527 [26]. However, we observed that the extracellular release of IL-6, enhanced by HHV-6A infection or DS-3201 treatment, further increased following EX-527 supplementation (Figure 4E,F), suggesting that the reduction of the intracellular fraction of IL-6 induced by EX-527 supplementation may be dependent on its higher release. Interestingly, despite the high release of IL-6 induced by HHV-6A or DS-3201 and its further increase with EX-527 supplementation, STAT3 705 tyrosine phosphorylation was reduced by these treatments in comparison to the untreated BCPAP (Figure 4G,H). This may correlate with the upregulation of Protein Tyrosine Phosphatase Non-Receptor Type 6 (PTPN6) STAT3 tyrosine phosphatase shown in Figure 3G,H [27,28], and could be a strategy that allows HHV-6A to limit the antiviral response, given the key role of STAT3 activation in the control of type I and III interferon (IFN) production [29].

As previous studies have shown that the depletion of SIRT1 increases EZH2 protein stability [30], here we evaluated whether EZH2 could be regulated by SIRT1 in BCPAP cells. The results shown in Figure 4G,H indicate that the SIRT1 inhibitor EX-527 downregulated EZH2, suggesting a reciprocal regulation between these two epigenetic enzymes in which EZH2 inhibits SIRT1 while the latter sustains EZH2 expression level.

### 3.4. EZH2-SIRT1 Axis Regulates c-Myc Expression and Cell Survival

**Figure 5:**
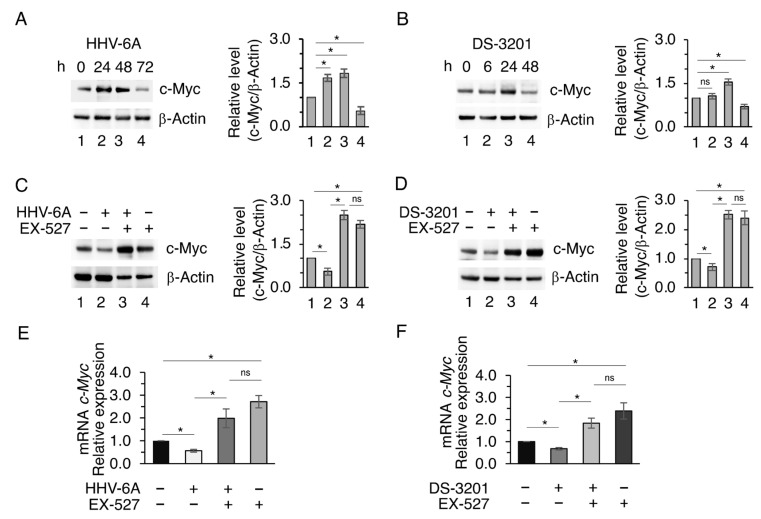
SIRT1 counteracts c-Myc upregulation in HHV-6A-infected or DS-3201-treated cells (**A**,**B**) Western blot analysis of c-Myc protein level in BCPAP cells infected with HHV-6A or treated with DS-3201 for the indicated times. (**C**–**F**) BCPAP cells were pre-treated or not with the SIRT1 inhibitor EX-527 for 1 h and then infected with mock or HHV-6A for 72 h or treated with DS-3201 for 48 h. (**C**,**D**) c-Myc protein level as assessed by Western blot and quantified by densitometry normalized to β-Actin, expressed as fold change relative to control. (**E**,**F**) *c-Myc* mRNA levels were measured by qRT-PCR, normalized to the reference gene *B2M*, and expressed relative to the control. All data are shown as mean ± S.D. from three independent experiments. *p* value * < 0.05, ns: non-significant.

Next, we evaluated whether EZH2 inhibition could also influence c-Myc whose expression has been previously shown to be upregulated in BCPAP infected by HHV-6A [9]. In Figure 5A,B, the time-course experiment shows that c-Myc expression level increased in HHV-6A-infected BCPAP as well as in cells treated by DS-3201, but decreased after long-term treatment. At this time of infection or DS-3201 treatment, SIRT1 was upregulated (Figure 3B,C), so we explored its role by using the EX-527 SIRT1 inhibitor. We found that this drug restored the expression level of c-Myc either in viral-infected BCPAP and in DS-3201-treated cells (Figure 5C,D), as well as in BCPAP undergoing EZH2 PROTAC MS177 treatment (Supplementary Figure S1B). The reduction in c-Myc was also observed at the mRNA level (Figure 5E,F), suggesting a transcriptional down-regulation of this molecule through the EZH2-SIRT1 axis downstream of HHV-6A infection. Most of the effects observed on HHV-6A-infected or valemetostat-treated BCPAP were reproduced on CAL-62, an anaplastic thyroid carcinoma cell line in which both treatments were also able to upregulate mutp53, SIRT1, IL-6, and c-Myc (Supplementary Figure S2).

**Figure 6:**
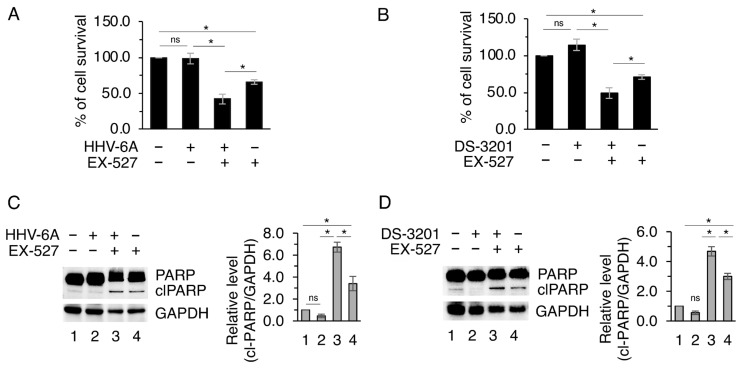
BCPAP cells’ survival is sustained by SIRT1 during HHV-6A infection or DS-3201 treatment. BCPAP cells were pre-treated or not with the SIRT1 inhibitor EX-527 for 1 h and then infected with mock or HHV-6A for 72 h or treated with DS-3201 for 48 h. (**A**,**B**) Cell viability was measured by a trypan blue exclusion assay, the histograms represent the mean  ±  S.D. of live cells as a percentage of untreated control cells, and (**C**,**D**) protein expression level of cleaved-(cl)PARP cleavage was evaluated by Western blot analysis. Histograms represent the densitometric analysis of the ratio of cl-PARP/GAPDH, as fold change relative to the control. All data are shown as mean ± S.D. from three independent experiments. *p* value * < 0.05, ns: non-significant.

Interestingly, SIRT1 inhibitor supplementation reduced BCPAP survival (Figure 6A,B) and induced the appearance of cleaved poly (ADP-ribose) polymerase (PARP) (Figure 6C,D), suggesting the occurrence of an apoptotic cell death. Therefore, the upregulation of SIRT1, consequent to EZH2 inhibition, may represent a strategy to promote the adaptation to stress caused by the persistent high expression of c-Myc and excessive IL-6 secretion, and to avoid the collapse of cell survival.

## 4. Discussion

Herpesviruses are well known to manipulate the host epigenetic machinery in order to establish persistence and promote pathogenic outcomes. This has been extensively documented for human cytomegalovirus [31] or Epstein–Barr virus [32,33], and our data indicate that HHV-6A operates through a similar strategy in thyroid cancer cells. HHV-6A has been reported to be associated with inflammatory diseases, including those affecting the thyroid tissue [3], and importantly, epigenetic alterations and chronic inflammation reinforce each other [34], generating a self-sustaining pathogenic circuit that may contribute to virus-associated diseases.

In this study, we found that HHV-6A infection of thyroid cancer cells resulted in downregulation of the histone methyltransferase EZH2. Although EZH2 is frequently overexpressed in cancer and classically associated with silencing of tumor suppressor genes, our findings support that the role of this methyltransferase may be more nuanced and context-dependent [11]. Indeed, in infected BCPAP cells, reduced EZH2 expression correlated with epigenetic deregulation is compatible with tumor progression, which is consistent with recent evidence indicating that loss of EZH2-mediated H3K27me3 derepresses inflammatory and stress-response genes, promoting oxidative stress and genomic instability [35]. Our results are also in line with reports showing that the reduced expression of EZH2 contributes to carcinogenesis in low-grade cancers [36].

Mechanistically, we found that the inhibition of EZH2 by valemetostat or by EZH2 PROTAC MS177 increased SIRT1 expression, establishing an EZH2–SIRT1 regulatory axis. This axis was critical for stabilization of mutant p53, as SIRT1-mediated deacetylation at lysine 382 enhanced mutp53 protein stability. Gain-of-function mutant p53 proteins are known drivers of tumor aggressiveness, and their accumulation provides a mechanistic link between viral epigenetic rewiring and oncogenic progression [37,38,39].

Mutp53 is known to enhance IL-6 production, and IL-6 signaling can reciprocally sustain mutp53 oncogenic activity, establishing a positive feedback loop that supports a tumor-promoting cellular state [40]. Here, we observed that EZH2 inhibition contributed to the release of IL-6, while SIRT1 counteracted this effect. Surprisingly, despite the high IL-6 release, STAT3 phosphorylation was reduced in viral-infected or DS-3201-treated BCPAP in the absence or in the presence of the SIRT1 inhibitor. This effect correlated with upregulation of PTPN6, the STAT3 phosphatase, and may be a strategy put in place by the virus to selectively dampen antiviral signaling while preserving pro-tumor inflammatory cues. Therefore, HHV-6A appears to be able to uncouple inflammatory tumor promotion from antiviral immune activation, consistent with a mechanism that may favor viral persistence.

Beyond mechanistic insights, our findings reveal a possible therapeutic vulnerability, raising the possibility that IL-6-targeting therapies such as Siltuximab or Tocilizumab could be explored against HHV-6A-associated thyroid cancers, particularly in combination with SIRT1 inhibitors.

Finally, here we observed that c-Myc, upregulated soon after EZH2 inhibition, was reduced by SIRT1, possibly as a mechanism to limit excessive oncogenic stress caused by persistent c-Myc activity. Consistently, pharmacological SIRT1 inhibition, which restored the expression of c-Myc, reduced cell viability, suggesting that the HHV-6A-induced epigenetic program balances proliferation and survival rather than maximizing oncogenic signaling.

## 5. Conclusions

Overall, our data support a model in which HHV-6A reshapes the host epigenetic landscape to promote a cellular state compatible with tumor progression and limit excessive oncogenic stress caused by persistent c-Myc upregulation, preserving cell survival. The virus also modulates IL-6 signaling and STAT3 activation, selectively remodeling the inflammatory pathways, which may help to escape from immune recognition. The novelty of this study lies in the identification of a mechanistic link between viral infection, chromatin remodeling, and oncogenic dependency, and raises the possibility that targeting IL-6 could be a therapeutic vulnerability of HHV-6A-associated thyroid cancer.

## Data Availability

The data presented in this study are available on request from the corresponding author.

## Supplementary Materials

The following supporting information can be downloaded at https://www.mdpi.com/article/10.3390/v18040409/s1. Figure S1: EZH2 degradation by MS177 increases intracellular IL-6 and downregulates c-Myc, effects counteracted by SIRT1 inhibition; Figure S2: HHV-6 infection or DS-3201 treatment induces similar molecular effects in CAL-62 cells.

## Abbreviations

The following abbreviations are used in this manuscript:

HHV-6A	Human herpesvirus 6 A
FTC	follicular carcinoma
ATC	anaplastic carcinoma
NF-κB	Nuclear Factor kappa-light-chain-enhancer of activated B cells
STAT3	Signal Transducer and Activator of Transcription 3
AP-1	Activating Protein-1
IL-6	Interleukin-6
EZH2	Enhancer of zeste 2
SIRT1	Sirtuin 1
PTM	Post-translational modification
HAT	Acetyltransferases
IFN	Interferon
PARP	Poly (ADP-ribose) polymerase
