# Supplementary material for: HHV-6A Drives Epigenetic Reprogramming via an EZH2–SIRT1 Axis to Sustain Mutant p53 and Reshape Oncogenic Inflammatory Signaling
Source: Viruses. 2026 Mar 26;18(4):409. doi: 10.3390/v18040409 (PMC13120135; doi:10.3390/v18040409)
Supplement: Supplementary file 1 [file viruses-18-00409-s001.zip › viruses-4195487-supplementary.pdf]

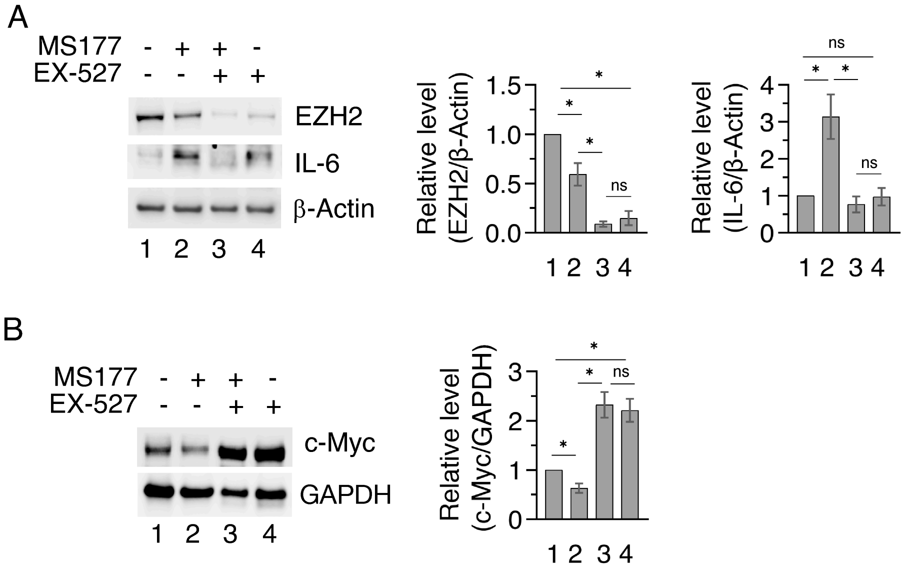

Figure S1

Figure S1. EZH2 degradation by MS177 increases intracellular IL-6 and downregulates c-Myc, effects counteracted by SIRT1 inhibition. (A, B) Western blot analysis of IL-6 and c-Myc protein level in BCPAP cells pre-treated or not with the SIRT1 inhibitor EX-527 for 1 h and then treated with treated with the EZH2 PROTAC degrader MS177 for 48 h. β-Actin or GAPDH were used as a loading control. Densitometric analyses were normalized to the appropriate control and expressed as fold change relative to the untreated condition. All data are shown as mean ± S.D. from three independent experiments. *p* value *< 0.05, ns: non-significant

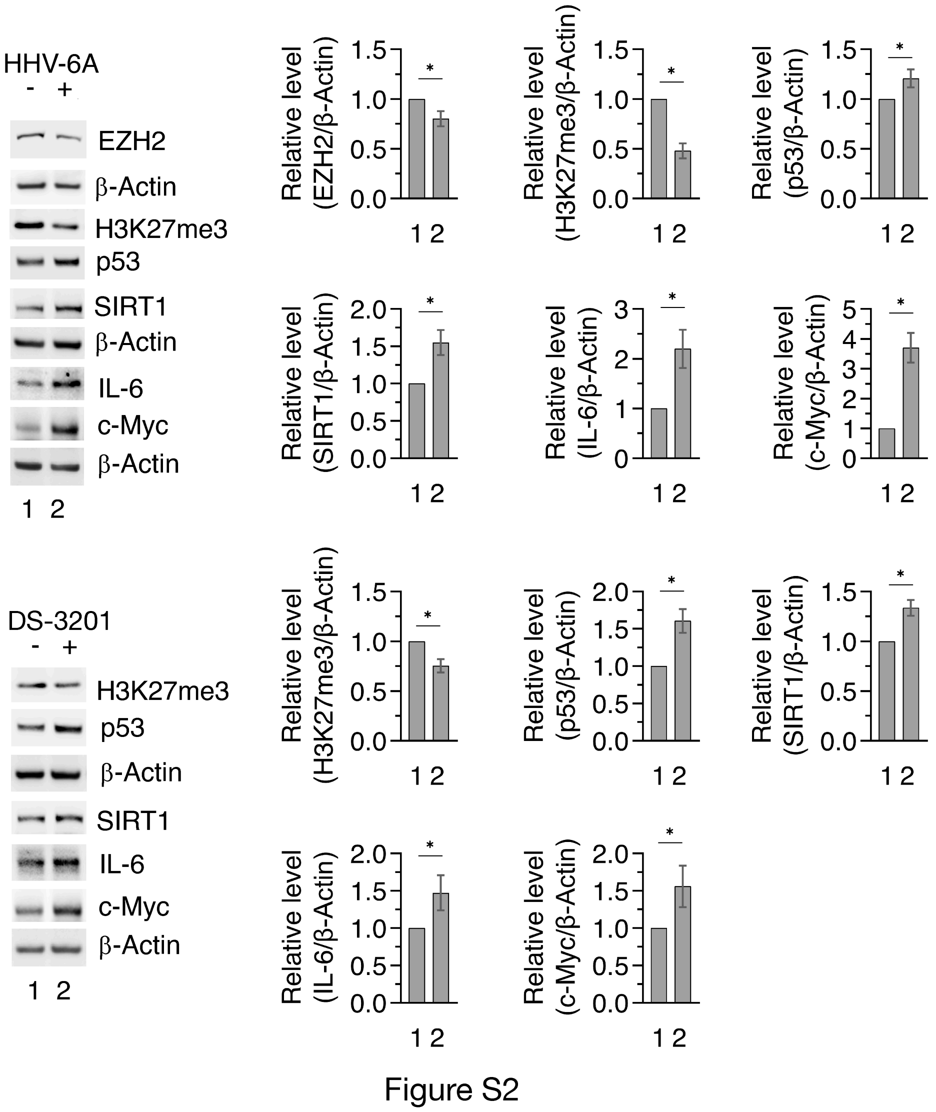

Figure S2. HHV-6 infection or DS-3201 treatment induces similar molecular effects in CAL-62 cells. Western blot analysis of EZH2, H3K27me3, mutp53, SIRT1, IL-6 and c-Myc protein levels in HHV-6A-infected or DS-3201-treated CAL-62 cells. β-Actin was used as a loading control. Densitometric analyses were normalized to the appropriate control and expressed as fold change relative to the untreated condition. All data are shown as mean ± S.D. from three independent experiments. *p* value *< 0.05.
